# Memory for neutral, emotional and trauma-related information in sexual abuse survivors

**DOI:** 10.1080/20008198.2018.1476439

**Published:** 2018-09-25

**Authors:** Marilyne Forest, Isabelle Blanchette

**Affiliations:** Département de Psychologie, Université du Québec à Trois-Rivières, Trois-Rivières, Québec, Canada

**Keywords:** Episodic memory, sexual abuse, neuropsychology, verbal memory, trauma, memoria episódica, abuso sexual, neuropsicología, memoria verbal, trauma, 情景记忆, 性虐待, 神经心理学, 言语记忆, 创伤, • Women victims of sexual abuse show poorer episodic memory, generally, compared to control participants.• This is true only for neutral and generally emotional information.• Victims do not show episodic memory deficits when content is related to sexual abuse, even if the content is not autobiographical.

## Abstract

Previous studies have shown that trauma-exposed individuals, including survivors of sexual abuse, show inferior performance in episodic memory tasks compared to non-exposed controls. This, however, has mainly been tested using neutral content. Our goal in this study was to determine whether this relative impairment in episodic memory extends to generally emotional and trauma-related content. Twenty-seven sexual abuse survivors and 27 control women participated in the study. They listened to stories with three content types (neutral, generally emotional and trauma-related) and performed a free-recall task immediately and 30 minutes later. Sexual abuse survivors showed poorer recall of neutral material compared to control participants. Lower recall was also observed for generally emotional content. However, importantly, there was no difference between groups in the recall of trauma-related content. The main novel contribution of this study is the demonstration that verbal episodic memory is not impaired for non-autobiographical trauma-related content in sexual abuse survivors. We discuss how this could be explained by personal relevance and attentional capture.

## Abstract (es)

Estudios previos han mostrado que individuos expuestos a trauma, incluyendo sobrevivientes de abuso sexual, mostraron un rendimiento inferior en tareas de memoria episódica comparados a controles no expuestos a trauma. Sin embargo, esto ha sido testeado principalmente usando contenidos neutrales. Nuestro objetivo en este estudio fue determinar si esta discapacidad relativa en la memoria episódica se extiende a los contenidos genéricamente emocionales y a los relacionados al trauma. Veintisiete mujeres sobrevivientes de abuso sexual y 27 mujeres control participaron en el estudio. Ellas escucharon historias de tres tipos de contenido (neutral, genéricamente emocional, relacionado al trauma) y ejecutaron una tarea de evocación espontánea inmediatamente y 30 minutos más tarde. Las sobrevivientes de abuso sexual mostraron una evocación más pobre de materiales neutrales, comparadas a las participantes control. Una evocación más baja fue también observada para contenidos genéricamente emocionales. Sin embargo, importantemente, no hubo diferencias entre grupos en la evocación de contenidos relacionados al trauma. La principal contribución original de este estudio es demostrar que la memoria episódica verbal no está afectada en cuanto a contenidos relacionados al trauma que no sean autobiográficos en las sobrevivientes de abusos sexual. Discutimos como esto puede ser explicado por relevancia personal y captura atencional.

## Abstract (zh)

先前的研究表明，创伤暴露的个体，包括性虐待幸存者，在情景记忆任务中的表现劣于非暴露控制。但是，这主要是使用中性内容进行测试的。我们在这项研究中的目标是确定情景记忆中的这种相对障碍是否可以延伸到一般情绪性和创伤相关内容。27名性虐待幸存者和27名对照妇女参加了这项研究。他们听取了三种内容类型的故事（中性的，一般情绪性的，与创伤有关的），在这之后和30分钟以后都完成了自由回忆任务。与对照者相比，性虐待幸存者对中性材料的回忆较差，对于一般的情绪内容也有较差的回忆。然而，重要的是，在回忆创伤相关内容方面，两组之间没有差异。这项研究的主要的新贡献是证明言语情景记忆不会损害性侵犯幸存者的非自传创伤相关内容。我们讨论了如何通过个人相关性和注意力捕获来解释这一点。

## 1. Introduction

Previous research has documented that trauma exposure is associated with alterations in memory function. This illustrates the important cognitive correlates of trauma exposure, and informs our understanding of the interaction between emotion and cognition. Few studies in this field have investigated the impact of the type of content to be remembered, in particular whether this content is trauma-related or neutral. In the current study, we investigated episodic memory in participants exposed to one form of trauma: sexual abuse. We examined memory for three types of content: neutral, generally emotional and emotional related to a sexual abuse (trauma-related). Our general goal was to determine whether the episodic memory impairments associated with trauma exposure – documented in previous research using neutral stimuli – are also observed with emotional and/or trauma-related content. The main goal of our study was to better understand the cognitive correlates of trauma exposure, but it may also have clinical implications, as remediation interventions need to be based on a precise characterization of the memory profile associated with different experiences. There are also implications for the legal system, where the testimony of trauma victims and witnesses must rely on memory.

**Figure 1.**
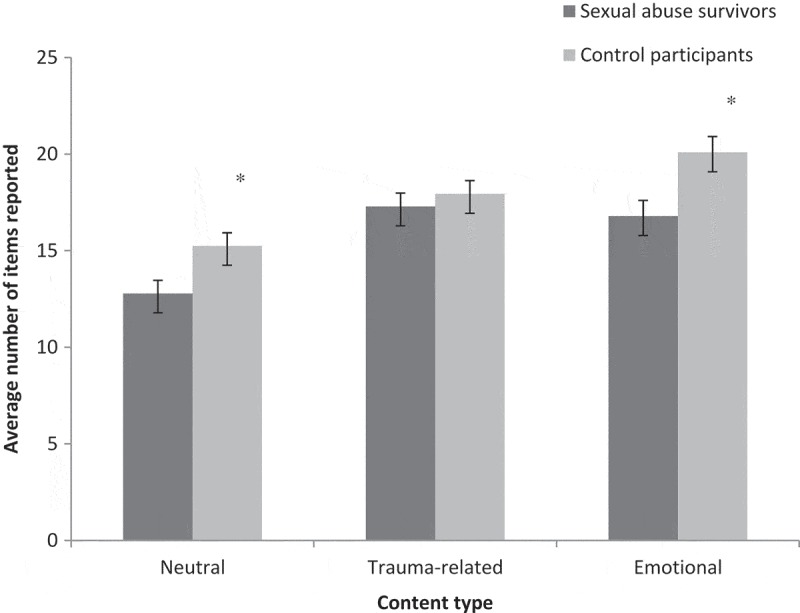
Average number of items reported (with SE) in the free-recall task for the different types of content by survivors of sexual abuse and control participants. *p < .05.

Based on current conceptions (Tulving, 2000; Tulving & Craik, 2000), we define episodic memory as involving the memory trace for an event that occurred at a specific time and place, and which can be explicitly recollected. Episodic memory is typically studied by presenting participants with stimuli to create a memory trace that is later reactivated (Picard, Eustache, & Piolino, 2009). Autobiographical memory, a related concept, is typically studied by asking participants to retrieve personal memories from their past (Picard et al., 2009; Piolino, 2005). Autobiographical memory has been proposed to be distinct from episodic memory in terms of personal relevance (Conway, 2002, 2005; Conway & Pleydell-Pearce, 2000). The recollection of autobiographical memories also significantly involves semantic memory (including knowledge of general events and general knowledge about the self).

### 1.1. Trauma, sexual abuse and memory function

Exposure to a potentially traumatic event, including sexual abuse, is associated with impairments in episodic memory. Rape survivors with post-traumatic stress disorder (PTSD) show poorer episodic memory than rape survivors without PTSD, and poorer memory than non-victims (Jenkins, Langlais, Delis, & Cohen, 1998). These impairments have been documented using different standardized memory tests – many presenting short stories followed by a free recall – including the California Verbal Learning Test and the Logical Memory subscale (e.g. Bremner, Vermetten, Afzal, & Vythilingam, 2004). A meta-analysis confirms that subsequent to an interpersonal trauma, individuals suffering from PTSD show verbal memory deficits compared to non-PTSD participants (Brewin, Kleiner, Vasterling, & Field, 2007). Trauma exposure may also be associated with lower performance on episodic memory tests, even independently of PTSD (Johnsen & Asbjørnsen, 2008; Rivera-Vélez, González-Viruet, Martínez-Taboas, & Pérez-Mojica, 2014).

Most studies of episodic memory in sexual abuse have evaluated memory using neutral stimuli. Emotional stimuli may produce different effects on memory. The emotion-superiority effect demonstrates that emotionally arousing events are more likely to be remembered than non-emotional ones (Kensinger & Schacter, 2008). Negative stimuli in particular are remembered more accurately, more vividly and with more detail than neutral events (Kensinger, 2007). This emotion-superiority effect has not always been observed, and varies according to a number of dimensions (Bennion, Ford, Murray, & Kensinger, 2013), but it is nevertheless a relatively robust phenomenon. How this influences episodic memory in relation to trauma experiences has not been extensively studied. Participants suffering from PTSD can show better recall for trauma-related material, compared to non-exposed controls (Paunovic, Lundh, & Ost, 2002). To our knowledge, it is not known whether this is the case for sexual abuse survivors in general, irrespective of PTSD. In this study, we investigate the possibility that episodic memory impairments in sexual abuse victims may be attenuated for stimuli semantically related to their trauma.

Autobiographical memory is better for trauma-related information than for other types of content. Porter and Peace (2007) interviewed adults about a personal trauma and a positive event that occurred to them. They reinterviewed these individuals 5 years later. Memories for the traumatic events were more consistent over this period of time compared to memories for the positive events; they tested participants' memory for details such the time of day, the clothes that participants were wearing and what the weather was like. In addition, trauma memories were more vivid and included more sensory details than positive memories. This study documents improved memory for trauma-related autobiographical information. This finding concurs with the conclusions of a review of the literature documenting a memory bias for traumatic autobiographical information in participants with PTSD (Tapia, Clarys, Isingrini, & El-Hage, 2007).

Studies of attention in trauma survivors also lead us to expect an improvement in memory for trauma-related stimuli. Trauma-exposed individuals preferentially allocate their attention to trauma-related stimuli. For instance, stimuli related to sexual abuse capture the attention of sexual abuse survivors (with PTSD) to a greater extent than neutral stimuli, and to a greater extent than healthy controls (Fleurkens, Rinck, & van Minnen, 2011). Attentional bias, because it enhances encoding, is typically associated with better memory (Kulas, Conger, & Smolin, 2003). Therefore, memory for trauma-related information may not be as impaired as memory for neutral content in sexual abuse survivors. This has not yet been directly investigated for episodic, non-autobiographical information.

Studies of implicit memory confirm the differential processing of trauma-related information. One prospective study (Ehring & Ehlers, 2011) found that participants who showed greater implicit memory for trauma-related material 2 weeks after surviving a motor vehicle accident showed more severe PTSD symptoms 6 months later. This was specific to accident-related stimuli; memory for general traffic (non-threatening) stimuli was not linked with later PTSD symptoms.

Other studies have examined episodic memory in relation to trauma using the Deese–Roediger–McDermott paradigm. In this task, participants encode lists of related words. In addition to correct recollection, the false recollection of critical lures – not presented but semantically related to the list – is measured. Women reporting histories of sexual abuse with PTSD show decreased correct recall and increased false recognition of critical lures compared to non-exposed, non-PTSD controls (Bremner, Shobe, & Kihlstrom, 2000). This study did not include trauma-related stimuli. Another study compared memory for trauma-related and neutral information, using the same paradigm but in war-exposed individuals (Brennen, Dybdahl, & Kapidžić, 2007). PTSD was associated with lower recall of trauma-related and neutral information, as well as increased false recall of trauma-related critical lures. This study confirms that memory for trauma-related and neutral information may be differentially affected in trauma-exposed individuals. This is what we examine with sexual abuse victims.

### 1.2. Overview of the study and hypotheses

Our primary interest in this study was to examine the memory correlates of trauma exposure, specifically sexual abuse. To our knowledge, no study has yet examined episodic memory for different types of non-autobiographical content in victims of sexual abuse, using a simple free-recall task. We examined memory for neutral, trauma-related and generally emotional stimuli.

We hypothesize that sexual abuse survivors would show poorer memory for neutral material compared to controls, based on previous studies of verbal memory and trauma exposure, including studies of sexual abuse victims. Our novel prediction was that this difference would be attenuated for content related to sexual abuse. We based this prediction on previous findings concerning preferential processing of trauma-related stimuli in attention and autobiographical memory. We included a generally emotional story to differentiate between the emotion-superiority effect and effects specifically related to trauma.

## 2. Method

### 2.1. Participants

Participants were recruited through advertisements on campus at the Université du Québec à Trois-Rivières, in the general community of Trois-Rivières and through support organizations for survivors of sexual abuse. The study was advertised as seeking ‘women who have and women who have not experienced sexual abuse’. Inclusion criteria were being female, and between 18 and 50 years old. Group membership (sexual abuse survivor, control) was determined using the questionnaires and interview (see later). Exclusion criteria, assessed in an initial telephone interview, were a history of neurological disorder, head injury, coma or loss of consciousness lasting more than 10 minutes, attention-deficit disorder with or without hyperactivity, learning disabilities, bipolar disorder, psychotic disorders, and alcohol or drug dependence and/or misuse. If participants met the criteria for major depressive disorder and the clinical evaluation (see later) suggested that these symptoms were more important than PTSD symptoms, participants were also excluded. The study obtained ethical approval from the Comité d’Éthique de la Recherche avec des Humains of the Université du Québec à Trois-Rivières (CER-11-175-06.13).

A total of 91 individuals volunteered for the study. Seven did not complete all parts of the memory task or failed to report their age. In the 84 remaining volunteers, there was a big age difference between groups. Women reporting experiences of sexual abuse were on average 12.0 years older than women in the control group [*t*(82) = 5.17, *p *< .001, *d *= 1.06]. To obtain two groups of equal size that were statistically equivalent for age, we included 54 women in the analyses, with equal numbers in the victim and control groups, matched for age.

There were 27 women in the sexual abuse survivor group. Participants were considered in this group if, during the clinical interview, they reported having experienced sexual abuse on the Life Events Checklist (LEC) (Gray, Litz, Hsu, & Lombardo, 2004) and if they reported having experienced at least one of the items related to sexual abuse on the Early Trauma Inventory – Short Form (ETI-SF) (Bremner, Bolus, & Mayer, 2007). We excluded the first item (‘touched on intimate parts in ways that made me uncomfortable’), which does not meet the legal definition for sexual abuse in the province of Québec, where the study was conducted. We adapted the ETI-SF to refer to entire life history, not exclusively events that occurred in childhood. Individuals in the control group did not report experiences of sexual abuse on the LEC or on the five items of the ETI-SF.

All participants had French as their first language. We present other participant characteristics in Table 1. The groups did not statistically differ in age or level of education.

### 2.2. Materials

#### 2.2.1. Memory task

The memory task used in the experiment was created by the first author and was based on the Logical Memory subscales of the Wechsler Memory Scale – Third Edition (WMS-III). The task consisted of three stories created by the first author: neutral, generally emotional and trauma-related. English translations of the stories are presented in the Appendix. The neutral story was about a woman watching television, the trauma-related story was about a woman experiencing date rape and the generally emotional story described a car accident. The stories were of equal lengths (85, 81 and 88 words, respectively). The nature of the stories was validated using an independent sample of 10 university students who evaluated arousal [from very low (1) to very high (9)] and valence [from very negative (1) to very positive (9)]. As intended, the arousal level of the neutral story [mean (*M*) = 1.40, *SD *= 0.70] was different from that of the generally emotional story (*M *= 5.60, *SD *= 2.68) [*t*(10.22) = − 4.80, *p* < .01] and the trauma-related one (*M *= 5.70, *SD *= 2.45) [*t*(10.45) = − 5.33, *p* < .01]. The generally emotional and the trauma-related stories did not differ significantly [*t*(18) = 0.09, *p* = .93]. The valence of the trauma-related story (*M *= 1.30, *SD *= 0.48) and of the generally emotional story (*M *= 1.30, *SD *= 0.68) did not differ statistically [*t*(18) = 0.00, *p* = 1.00], but both differed from the neutral story (*M *= 5.20, *SD *= 0.79) [*t*(18) = 11.88, *p* < .01 and *t*(18) = 13.33, *p* < .01, respectively].

We developed a coding scheme to quantify the free recall of participants. Twenty-eight items corresponding to informational elements from the story were listed for each story. The items for the neutral story are presented as an example in the Appendix (the complete material is available from the authors upon request). One point was allocated for the recall of each item. Points were summed to produce total immediate recall and delayed recall scores for each story. While coding, we noted all instances of false alarms (incorrectly recalling information that was not presented), but these were too few to analyse (< 10 in total).

An index of the reliability of the memory task is provided by the correlation between immediate and delayed (30 minute) recall, which we examined separately for the three content types, and for the two groups of participants. All six correlations were significant (*p* < .001) and were all higher than *r *= .80, with only one exception for the trauma-related stories in the victim group (*r *= .70, *p *< .001).

#### 2.2.2. Questionnaires

The French version of the ETI-SF (Bremner et al., 2007) was administered to participants to assess experiences of sexual abuse. This questionnaire includes six questions about possible experiences of sexual abuse. We adapted the instruction to refer to entire life history, rather than childhood experiences exclusively. As mentioned previously, we did not consider answers to one of the questions (item 1), which does not unambiguously meet the legal criterion for sexual abuse.

A French version of the Impact of Event Scale – Revised (IES-R) (Brunet, St-Hilaire, Jehel, & King, 2003) was filled out by participants to evaluate the level of subjective distress and affective consequences associated with a potential trauma. Participants in the survivor group answered these questions in relation to their experience(s) of sexual abuse and controls answered in relation to their most emotional event. Participants also filled in the French version of the State–Trait Anxiety Inventory (STAI) and a French version of the LEC) (Gray et al., 2004), to evaluate the number of potentially traumatic events experienced over the course of their lifespan.

A French version of the Clinician Administered PTSD Scale for DSM-IV (CAPS) (Saint-Onge, n.d.) was administered to evaluate the presence and severity of PTSD symptoms. For participants in the survivor group, questions were related to their experience(s) of sexual abuse. For controls, questions concerned their most emotional event. We used the habitual scoring procedure; to meet the diagnostic criterion, participants had to report at least one re-experiencing, three avoidance and two arousal symptoms in the past month, with a minimal intensity of 2. A French version of the Anxiety Disorders Interview Schedule for DSM-IV – Lifetime version (ADIS-IV-L) was administered to evaluate major depressive disorder. If the depressive symptoms were more important than the PTSD symptoms, participants were not included in the analyses.

### 2.3. Procedure

The procedure included three main parts: a telephone interview, the clinical interview and the cognitive tasks. The telephone interview served to describe the study and evaluate inclusion and exclusion criteria. We also asked participants whether they had been involved in a serious road accident in the past. The clinical interview, which included the CAPS and the ADIS, was administered by a Clinical Psychology doctorate student. Participants came back to the laboratory within 1 week for the cognitive tasks, before which they filled in the ETI-SF, which determined group membership. In addition to the memory task reported here, the procedure included other tasks evaluating reasoning and attention. This study was part of a larger project which investigates the impact of highly emotional events (sexual abuse and car accidents) on cognitive function. The other cognitive tasks were performed during the delay between immediate and delayed recall.

During the memory task, each participant listened to the three stories, with presentation order randomized across participants. The three stories were recorded with VLC media player and read by the same female narrator. Participants wore headphones and were instructed to listen to the stories and to try to remember as much information as possible. After each story, participants were asked to recall as many details as possible about the story. They did this recall orally, speaking into a microphone while their answers were recorded. They were allowed a maximum of 1 minute. Thirty minutes later, participants undertook the free recall again but the time allowed was a maximum of 1.5 minutes.

The cognitive tasks lasted on average 1 hour and 30 minutes. Participants were awarded compensation of $30.

## 3. Results

### 3.1. Characteristics of participants

Table 1 presents the participants’ characteristics. Sexual abuse survivors and control participants differed on the number of potentially traumatic events experienced, trait anxiety (STAI) and the affective consequences associated with an emotional event (IES-R). Among sexual abuse survivors, seven participants had been diagnosed with PTSD in the past, whereas none of the control participants had. Sexual abuse survivors showed higher levels of PTSD symptoms overall (CAPS). Table 2 presents the correlations between the affective consequences associated with an emotional event (IES-R), the number of sexual abuse experience(s) (ETI-SF) and other variables.

**Table 1.** Participants’ sociodemographic and psychometric characteristics.

	Sexual abuse survivors (*n* = 27)	Controls (*n* = 27)	*χ*^2^	*p*
Number of participants currently in psychotherapy	7 (26%)	1 (4%)	5.28	< .05
Number of participants taking psychotropic medication	3 (11%)	2 (7%)	0.22	.64
Number of participants reporting a psychopathology	7 (26%)	4 (15%)	1.03	.31
Number of participants reporting car accident(s)	14 (52%)	24 (48%)	0.74	.78
	*M* (*SD*)	*M* (*SD*)	*t*	*p*
Age (years)	29.3 (8.8)	34.0 (12.9)	1.56	.12
Years of education	15.3 (1.3)	14.9 (1.5)	1.27	.21
Impact of event (IES-R)	22.37 (19.95)	5.63 (8.82)	3.99	< .01
Anxiety (STAI)	43.07 (10.95)	35.22 (7.08)	3.13	< .01
Potentially traumatic events (LEC)	4.18 (2.13)	2.11 (2.13)	3.11	< .01
PTSD symptoms (CAPS)	20.44 (13.94)	7.04 (8.26)	4.29	< .01
Sexual abuse (ETI-SF)	2.63 (1.54)	0.00 (0.0)	8.45	< .01

IES-R, Impact of Event Scale – Revised; STAI, Spielberger State–Trait Anxiety Inventory; LEC, Life Events Checklist; PTSD, post-traumatic stress disorder; CAPS, Clinician Administered PTSD Scale; ETI-SF, Early Trauma Inventory – Short Form.

**Table 2.** Correlations between the clinical measures.

	IES-R	LEC	ETI-SF	STAI	CAPS total	Re-experiencing	Avoidance
Distress related to emotional event (IES-R)							
Number of potentially traumatic events (LEC)	.32*						
Experiences of sexual abuse (ETI-SF)	.49**	.63**					
Trait Anxiety (STAI)	.48**	.15	.27*				
PTSD symptoms (CAPS total)	.74**	.32*	.49**	.48**			
CAPS – Re-experiencing	.52**	.18	.36**	.16	.78**		
CAPS – Avoidance	.60**	.37*	.47**	.41**	.85**	.57**	
CAPS – Arousal	.69**	.21	.37**	.56**	.84**	.48**	.53**

IES-R, Impact of Event Scale – Revised; LEC, Life Events Checklist; ETI-SF, Early Trauma Inventory – Short Form; STAI, Spielberger State–Trait Anxiety Inventory; CAPS, Clinician Administered PTSD Scale; PTSD, post-traumatic stress disorder.

*p < .05, **p < .01.

### 3.2. Memory task

Figure 1 presents the number of items recalled by the two groups for the generally emotional, neutral and trauma-related stories. A 3 (Content type: neutral, generally emotional and trauma-related) by 2 (Group: sexual abuse survivors and controls) by 2 (Time of retrieval: immediate and delayed) mixed analysis of variance (ANOVA) was conducted to compare the number of items recalled. A significant interaction was observed between Group and Content [*F*(2, 104) = 3.17, *p *< .05, *n*^2^_p_ = .06]. Post-hoc tests, correcting for the number of comparisons, were conducted to evaluate the difference between the two groups for each Content type separately (averaging over immediate and delayed recall). Sexual abuse survivors recalled less information about the neutral story than controls [*t*(52) = 2.57, *p* < .01, *d *= .70]. Survivors also recalled significantly less information about the generally emotional story than controls [*t*(52) = 2.85, *p* < .01, *d *= .76]. However, scores for the trauma-related story did not significantly differ between the two groups [*t*(52) = 0.66, *p* = .50, *d *= .18]. The interaction therefore stemmed from a greater difference between survivors and controls for the neutral and generally emotional content compared to the trauma-related content. All three main effects were significant [Group, *F*(1, 52) = 6.67, *p *< .01, *n*^2^_p_ = .12; Content type, *F*(2, 52) = 38.14, *p *< .01, *n*^2^_p_ = .42; and Time, *F*(2, 52) = 39.35, *p *< .01, *n*^2^_p_ = .43]. Participants recalled a greater number of items in immediate recall (*M* = 17.44, *SE* = 0.41) compared to delayed recall (*M* = 15.95, *SE* = 0.45). No other effects were significant; Time did not interact with Content type or Group.

Recall scores for the neutral story were negatively correlated with the number of traumatic events reported (LEC, *r *= –.30, *p *< .05). Similarly, recall of the generally emotional story was also negatively related to the LEC (*r *= –.40, *p *< .01). Recall of the trauma-related story was not correlated with any of the symptoms (IES-R, CAPS) or past experiences scales (LEC). These analyses were conducted over the entire sample.

When restricting analyses to the group of sexual abuse victims (*n* = 27), none of the correlations between the memory measures and the questionnaire/clinical measures was significant. Furthermore, comparing victims who met the diagnostic criterion on the CAPS (*n* = 7) to those who did not (*n* = 20) showed no significant differences in memory for neutral [*t*(25) = 0.56, *p *= .58], generally emotional [*t*(25) = 1.45, *p *= .16] or trauma-related items [*t*(25) = 1.15, *p *= .26].

## 4. Discussion

The main goal of this study was to determine the impact of content type on the episodic memory of sexual abuse survivors. Our results confirmed that victims of sexual abuse show poorer episodic memory for neutral information compared to non-exposed controls. This impairment is also observed for generally emotional content. Importantly, however, and in line with our hypotheses, victims did not show an impairment in memory for trauma-related content. In addition to these main findings, we found a general link between potentially traumatic events and memory: participants who reported a greater number of potentially traumatic events (including sexual abuse) had worse memory for neutral and generally emotional information.

The finding that episodic memory for trauma-related content was not significantly impaired among a population of sexual abuse survivors is novel and important. Studies of autobiographical memory show that experiences of sexual abuse are recalled more vividly and with more detail than positive events, and even more vividly than other potentially traumatic memories (Porter & Peace 2007). More generally, increased memory for trauma-related information, in the form of flashbacks and intrusive memories, is a cardinal feature of PTSD and trauma (Brewin, 2015; van Harmelen, Elzinga, Kievit, & Spinhoven, 2011). Our results suggest that this increased memory extends to stimuli that are semantically related to these personal experiences but that are not autobiographical. While not autobiographical, the trauma-related content presented in our task may nevertheless have been encoded as more personally relevant than the neutral content. Encoding information in a self-relevant way is generally associated with better memory (Symons & Johnson, 1997), an effect that may result from better strategies or better organization in the encoding the self-relevant material. When women with experience(s) of sexual abuse encode abuse-related material, they may include it in more elaborate mnemonic networks. The attentional bias for trauma-related information among sexual abuse survivors (Fleurkens et al., 2011) can also help to explain the reduced memory impairment for sexual abuse content that we observed. During encoding, trauma-related material may thus benefit from a number of memory-enhancing factors: attentional prioritization, attribution of additional cognitive resources and encoding in relation to extended memory networks.

One previous study of episodic memory showed effects analogous to the one we observed (Golier, Yehuda, Lupien, & Harvey, 2003). In that study, Holocaust survivors suffering from PTSD recalled less neutral information than controls, in a paired-associates test. As in our study, this memory impairment was reduced for trauma-related words, in this case Holocaust-related words. One difference from our study is that Holocaust survivors not suffering from PTSD did not differ from non-exposed controls, whereas we found a difference as a function of trauma exposure per se. One possible explanation for this discrepancy is time since exposure; the average time since focal trauma for the Holocaust survivors was approximately 53.9 years, which is longer than the average time since trauma in our study (given that the average age in the victim group was 29.3 years old). The difference in findings may indicate a longer lasting effect of psychopathology compared to trauma exposure.

Previous studies provided conflicting findings concerning the impact of content type on memory in trauma victims. In two studies using a retrieval-induced forgetting paradigm, trauma-related words did not lead to different performance compared to neutral words (Amir, Badour, & Freese, 2009; Blix & Brennen, 2012). In one of these studies, the sample included individuals exposed to diverse traumas, and the trauma-related stimuli may not have been sufficiently personally relevant to induce differential processing (Amir, Badour, & Freese, 2009). In the other study, the results failed to replicate the basic retrieval-induced forgetting effect expected (except in reaction times) (Blix & Brennen, 2012). Another study, which used a slightly different paradigm (directed forgetting), found that sexual abuse victims were more likely to recall trauma-related words they had been instructed to forget, compared to non-exposed participants; however, this was not the case for words they had been instructed to recall (Blix & Brennen, 2011). Overall, previous findings have thus been equivocal concerning the effects of content type on memory in trauma victims. Conflicting findings may partly result from the diversity in methods used. Our results, using a simple free-recall paradigm, show that memory for trauma-related content does differ from memory for neutral information.

The finding that sexual abuse survivors recalled less neutral information than non-exposed controls confirms the conclusion of other studies (Bremer et al. 2004; Johnsen & Asbjørnsen, 2008; Rivera-Vélez et al., 2014). A majority of these studies used the Wechsler Logical Memory Scale, the test on which we based on our methodology. This test includes an immediate and a 30 minute delayed recall. Our results thus concur with those of studies looking at memory over similar intervals. It should be noted that most studies, including ours, have therefore documented the correlates of trauma experiences on episodic memory at relatively short intervals. Because of the important and complex consolidation processes that occur over hours, days and months following encoding, future studies will need to include longer retention intervals to provide a more complete picture of the impact of trauma on episodic memory.

In addition to specific experiences such as sexual abuse, previous studies suggest that the accumulation of traumatic experiences may be negatively associated with verbal memory (Nixon, Nishith, & Resick, 2004). Our study provides a similar result: the total number of potentially traumatic events reported by women (including sexual abuse) was negatively correlated with memory for neutral content, as well as generally emotional material.

Our study has some limitations. The procedure may have induced differences in the state of mind of participants while they performed the memory task. Participants answered six questions concerning their history of abuse, at the beginning of the session. These questions may have primed abuse-related thoughts in victims. Another limitation is that we did not collect information concerning the time elapsed since the abuse. It is therefore not possible to compare the effects of childhood sexual abuse and abuse experienced in adulthood. Future studies should conduct more detailed psychometric assessments to match groups on affective and cognitive characteristics. Our study, like all studies of trauma experiences, is correlational. Differences between the groups, other than past history of sexual abuse, could account for the results (for example, differences in cognitive abilities or experience of psychotherapy). Finally, the terms ‘impairment’ and ‘deficit’ need to be used with caution. We speak of a memory impairment for neutral information, but this impairment is relative, and simply indicates lower performance in the victim group compared to the non-exposed control group. This should not be understood to mean that there is a pathological level of memory function. There are no normative data for the task we developed, and the goal is not to draw conclusions about normal or abnormal function.

Despite these limitations, this study has improved our understanding of the association between trauma exposure and cognitive function. Our results reveal that episodic memory impairments are not universal; they may be present for neutral and generally emotional stimuli, but not for trauma-related content. This is important to qualify conclusions concerning memory alterations related to trauma and may have important implications for legal settings; for instance, where it should not be assumed that trauma victims will have poorer memory in general.

## Appendix

Stories used in the episodic memory task

**Trauma-related**

*Original French version*: Un samedi soir, Camille, de Québec, était à une soirée pour la fête d’un ami. Elle avait bu beaucoup d’alcool. Elle était seule dans la chambre de son ami pour se reposer, car elle avait mal au cœur. Jasmin, une connaissance, est venu la rejoindre et a commencé à la caresser. Elle lui a dit d’arrêter et a essayé de s’en aller, en vain. Il l’a forcée à avoir une relation sexuelle. Le lendemain, elle s’est remémoré l’événement et était bouleversée.

*Translation*: On a Saturday evening, Camille, from Québec, was at a party for a friend’s birthday. She had drunk a lot of alcohol. She was by herself in her friend’s room, because she was feeling nauseous. Jasmin, an acquaintance, came into the room and started caressing her. She told him to stop and tried to get away but she couldn’t. He forced her to have sex. The next day, she recalled the event and was distressed.

**Generally emotional**

*Original French version*: Un vendredi soir, Rébecca, de Montréal, s’en allait visiter ses parents en voiture avec ses enfants. Ils allaient passer la journée du lendemain au zoo. Un conducteur en état d’ébriété a dévié de sa voie. Il y a eu un choc terrible. Rebecca a entendu ses enfants pleurer et crier. Elle a ensuite perdu conscience. À son réveil à l’hôpital, elle a appris la mort de ses enfants des suites de blessures à la tête. Elle était bouleversée.

*Translation*: On a Friday evening, Rebecca, from Montréal, was going to visit her parents, by car, with her children. They were going to spend the following day at the zoo. An intoxicated driver deviated from his lane. There was a tremendous crash. Rebecca heard her children cry and scream. Then she fainted. When she woke up in the hospital, she learned that her children had died from head injuries. She was distressed.

Neutral

*Original French version*: Un mardi soir, Émilie, de Trois-Rivières, regardait la télévision tout en préparant son souper. Elle écoutait le bulletin de nouvelles. Le présentateur parlait de la hausse du prix de l’essence à cause du début des vacances. L’animatrice météo a annoncé les prévisions de cette semaine. La journée du mercredi s’annonce ensoleillée avec une température de 30°C. Les prévisions météo annoncent de la pluie pour jeudi, mais le beau temps sera de retour vendredi. Émilie a ensuite écouté un quiz télévisé.

*Translation*: On a Tuesday evening, Emily, from Trois-Rivières, was watching television while preparing her dinner. She was watching the news. The news reporter was talking about the increase in petrol prices, because of the start of the holidays. The weather forecaster was announcing the forecast for the week. Wednesday was going to be sunny with a temperature of 30°C. The forecast predicted rain for Thursday, but that there should be nice weather on Friday. Emily then watched a game show.

Coding scheme:

**Table:**

Item	
Un mardi	
soir,	
Émilie	
de Trois-Rivières	
regardait la télévision	
tout en préparant son souper.	
Elle écoutait le bulletin de nouvelles,	
le présentateur	
parlait de la hausse du prix	
de l’essence	
à cause du début des vacances.	
L’animatrice météo	
a annoncé les prévisions	
de cette semaine.	
La journée	
du mercredi	
s’annonce ensoleillée	
avec une température de 30°C.	
Les prévisions météo	
annoncent de la pluie	
pour jeudi,	
mais le beau temps	
sera de retour	
vendredi.	
Émilie	
a ensuite écouté	
un quiz télévisé	

## References

[CIT0001] Amir N., Badour C. L., & Freese B. (2009). The effect of retrieval on recall of information in individuals with posttraumatic stress disorder. *Journal of Anxiety Disorders*, 23(4), 535–10. 19070459 10.1016/j.janxdis.2008.10.012 PMC3808957

[CIT0002] Bennion K. A., Ford J. H., Murray B. D., & Kensinger E. A. (2013). Oversimplification in the study of emotional memory. *Journal of the International Neuropsychological Society: JINS*, 19(9), 953–961. 24007950 10.1017/S1355617713000945 PMC3955981

[CIT0003] Blix I., & Brennen T. (2011). Intentional forgetting of emotional words after trauma: A study with victims of sexual assault. *Frontiers in Psychology*, 2(SEP), 1–8. 21994497 10.3389/fpsyg.2011.00235 PMC3182753

[CIT0004] Blix I., & Brennen T. (2012). Retrieval-induced forgetting after trauma: A study with victims of sexual assault. *Cognition and Emotion*, 26(2), 321–331. 21574076 10.1080/02699931.2011.570312

[CIT0005] Bremner J. D., Bolus R., & Mayer E. A. (2007). Psychometric properties of the Early Trauma Inventory-Self-Report. *Journal of Nervous and Mental Disease*, 195(3), 211–218. 17468680 10.1097/01.nmd.0000243824.84651.6c PMC3229091

[CIT0006] Bremner J. D., Shobe K. K., & Kihlstrom J. F. (2000). False memories in women with self-reported childhood sexual abuse: An empirical study. *Psychological Science*, 11(4), 333–337. 11273395 10.1111/1467-9280.00266

[CIT0007] Bremer J. D., Vermetten E., Afzal N., & Vythilingam M. (2004). Deficits in verbal declarative memory function in women with childhood sexual abuse-related posttraumatic stress disorder. *Journal of Nervous and Mental Disease*, 192(10), 643–649. 15457106 10.1097/01.nmd.0000142027.52893.c8

[CIT0008] Brennen T., Dybdahl R., & Kapidžić A. (2007). Trauma-related and neutral false memories in war-induced posttraumatic stress disorder. *Consciousness and Cognition*, 16(4), 877–885. 16901721 10.1016/j.concog.2006.06.012

[CIT0009] Brewin C. R. (2015). Re-experiencing traumatic events in PTSD: New avenues in research on intrusive memories and flashbacks. *European Journal of Psychotraumatology*, 6. doi:10.3402/ejpt.v6.27180 PMC4439411 25994019

[CIT0010] Brewin C. R., Kleiner J. S., Vasterling J. J., & Field A. P. (2007). Memory for emotionally neutral information in posttraumatic stress disorder: A meta-analytic investigation. *Journal of Abnormal Psychology*, 116(3), 448–463. 17696700 10.1037/0021-843X.116.3.448

[CIT0011] Brunet A., St-Hilaire A., Jehel L., & King S. (2003). Validation of a French version of the impact of event Scale-Revised. *The Canadian Journal of Psychiatry/La Revue Canadienne de Psychiatrie*, 48(1), 56–61. 10.1177/070674370304800111 12635566

[CIT0012] Conway M. A. (2002). Sensory-perceptual episodic memory and its context: Autobiographical memory In Baddeley A., Aggleton J. P., Conway M. A., Baddeley A., Aggleton J. P., & Conway M. A. (Eds.), *Episodic memory: New directions in research* (pp. 53–70). New York, NY: Oxford University Press.

[CIT0013] Conway M. A. (2005). Memory and the self. *Journal of Memory and Language*, 53(4), 594–628.

[CIT0014] Conway M. A., & Pleydell-Pearce C. W. (2000). The construction of autobiographical memories in the self-memory system. *Psychological Review*, 107(2), 261–288. 10789197 10.1037/0033-295x.107.2.261

[CIT0015] Ehring T., & Ehlers A. (2011). Enhanced priming for trauma-related words predicts posttraumatic stress disorder. *Journal of Abnormal Psychology*, 120(1), 234–239. 21058753 10.1037/a0021080 PMC3073491

[CIT0016] Fleurkens P., Rinck M., & van Minnen A. (2011). Specificity and generalization of attentional bias in sexual trauma victims suffering from posttraumatic stress disorder. *Journal of Anxiety Disorders*, 25(6), 783–787. 21531116 10.1016/j.janxdis.2011.03.014

[CIT0017] Golier J. A., Yehuda R., Lupien S. J., & Harvey P. D. (2003). Memory for trauma-related information in Holocaust survivors with PTSD. *Psychiatry Research*, 121(2), 133–143. 14656448 10.1016/s0925-4927(03)00120-3

[CIT0018] Gray M. J., Litz B. T., Hsu J. L., & Lombardo T. W. (2004). Psychometric properties of the life events checklist. *Assessment*, 11(4), 330–341. 15486169 10.1177/1073191104269954

[CIT0019] Jenkins M. A., Langlais P. J., Delis D., & Cohen R. (1998). Learning and memory in rape victims with posttraumatic stress disorder. *The American Journal of Psychiatry*, 155(2), 278–279. 9464211 10.1176/ajp.155.2.278

[CIT0020] Johnsen G. E., & Asbjørnsen A. E. (2008). Consistent impaired verbal memory in PTSD: A meta-analysis. *Journal of Affective Disorders*, 111(1), 74–82. 18377999 10.1016/j.jad.2008.02.007

[CIT0021] Kensinger E. A. (2007). Negative emotion enhances memory accuracy: Behavioral and neuroimaging evidence. *Current Directions in Psychological Science*, 16(4), 213–218.

[CIT0022] Kensinger E. A., & Schacter D. L. (2008). Memory and emotion In Lewis M., Haviland-Jones J. M., & Barrett L. F. (Eds.), *Handbook of emotions* (3rd ed., pp. 601–617). New York, NY: Guilford Press.

[CIT0023] Kulas J. F., Conger J. C., & Smolin J. M. (2003). The effects of emotion on memory: An investigation of attentional bias. *Journal of Anxiety Disorders*, 17(1), 103–113. 12464292 10.1016/s0887-6185(02)00177-9

[CIT0024] Nixon R. D. V., Nishith P., & Resick P. A. (2004). The accumulative effect of trauma exposure on short-term and delayed verbal memory in a treatment-seeking sample of female rape victims. *Journal of Traumatic Stress*, 17(1), 31–35. 15027790 10.1023/B:JOTS.0000014673.02925.db PMC2977921

[CIT0025] Paunovic N., Lundh L. G., & Ost L. G. (2002). Attentional and memory bias for emotional information in crime victims with acute posttraumatic stress disorder (PTSD). *Journal of Anxiety Disorders*, 16(6), 675–692. 12405525 10.1016/s0887-6185(02)00136-6

[CIT0026] Picard L., Eustache F., & Piolino P. (2009). De la mémoire épisodique á la mémoire autobiographique : Approche développementale. *Annee Psychologique*, 109(2), 197–236.

[CIT0027] Piolino P. (2005). *Neuropsychology of autobiographical memor. Cortex 39*(4–5), 687–728. 10.1016/s0010-9452(08)70860-8 14584549

[CIT0028] Porter S., & Peace K. A. (2007). The scars of memory of traumatic and positive emotional memories in adulthood. *Psychological Science*, 18(5), 435–441. 17576284 10.1111/j.1467-9280.2007.01918.x

[CIT0029] Rivera-Vélez G. M., González-Viruet M., Martínez-Taboas A., & Pérez-Mojica D. (2014). Post-traumatic stress disorder, dissociation, and neuropsychological performance in Latina victims of childhood sexual abuse. *Journal of Child Sexual Abuse: Research, Treatment, & Program Innovations for Victims, Survivors, & Offenders*, 23(1), 55–73. 10.1080/10538712.2014.864746 24393090

[CIT0030] Symons C. S., & Johnson B. T. (1997). The self-reference effect in memory: A meta-analysis. *Psychological Bulletin*, 121(3), 371–394. 9136641 10.1037/0033-2909.121.3.371

[CIT0031] Tapia G., Clarys D., Isingrini M., & El-Hage W. (2007). Mémoire et émotion dans le trouble de stress post-traumatique (TSPT) [Memory and emotion in post-traumatic stress disorder]. *Canadian Psychology/Psychologie Canadienne*, 48(2), 106–119.

[CIT0032] Tulving E. (2000). Concepts of memory In E. Tulving, F. M. Craik (Eds.), *The Oxford handbook of memory* (pp. 33-43). New York, NY: Oxford University Press.

[CIT0033] Tulving E., & Craik F. M (2000). *The Oxford handbook of memory*. New York, NY: Oxford University Press.

[CIT0034] van Harmelen A.-L., Elzinga B., Kievit R., & Spinhoven P. (2011). Intrusions of autobiographical memories in individuals reporting childhood emotional maltreatment. *European Journal of Psychotraumatology*, 2(1), 7336. 10.3402/ejpt.v2i0.7336 PMC3402144 22893818

